# Biomarkers of Response and Resistance to CDK4/6 Inhibitors in Breast Cancer: Hints from Liquid Biopsy and microRNA Exploration

**DOI:** 10.3390/ijms232314534

**Published:** 2022-11-22

**Authors:** Eriseld Krasniqi, Frauke Goeman, Claudio Pulito, Alina Catalina Palcau, Ludovica Ciuffreda, Francesca Sofia Di Lisa, Lorena Filomeno, Maddalena Barba, Laura Pizzuti, Federico Cappuzzo, Giuseppe Sanguineti, Marcello Maugeri-Saccà, Gennaro Ciliberto, Maurizio Fanciulli, Giovanni Blandino, Patrizia Vici

**Affiliations:** 1 Division of Medical Oncology 2, IRCCS Regina Elena National Cancer Institute, 00144 Rome, Italy; 2 SAFU Unit, IRCCS Regina Elena National Cancer Institute, 00144 Rome, Italy; 3 Translational Oncology Research Unit, Department of Research, Advanced Diagnostic and Technological Innovation, IRCCS Regina Elena National Cancer Institute, 00144 Rome, Italy; 4 Phase IV Clinical Studies Unit, IRCCS Regina Elena National Cancer Institute, 00144 Rome, Italy; 5 Department of Radiation Oncology, IRCCS Regina Elena National Cancer Institute, 00144 Rome, Italy; 6 Clinical Trial Center, Biostatistics and Bioinformatics, IRCCS Regina Elena National Cancer Institute, 00144 Rome, Italy; 7 Scientific Direction, IRCCS Regina Elena National Cancer Institute, 00144 Rome, Italy

**Keywords:** breast cancer, CDK4/6 inhibitors, liquid biopsy, microRNA, palbociclib, abemaciclib, ribociclib

## Abstract

New evidence on the impact of dysregulation of the CDK4/6 pathway on breast cancer (BC) cell proliferation has led to the development of selective CDK4/6 inhibitors, which have radically changed the management of advanced BC. Despite the improved outcomes obtained by CDK4/6 inhibitors, approximately 10% of tumors show primary resistance, whereas acquired resistance appears to be an almost ubiquitous occurrence, leading to treatment failure. The identification of differentially expressed genes or genomic mutational signatures able to predict sensitivity or resistance to CDK4/6 inhibitors is critical for medical decision making and for avoiding or counteracting primary or acquired resistance against CDK4/6 inhibitors. In this review, we summarize the main mechanisms of resistance to CDK4/6 inhibitors, focusing on those associated with potentially relevant biomarkers that could predict patients’ response/resistance to treatment. Recent advances in biomarker identification are discussed, including the potential use of liquid biopsy for BC management and the role of multiple microRNAs as molecular predictors of cancer cell sensitivity and resistance to CDK4/6 inhibitors.

## 1. Introduction

Cyclin-dependent kinases 4 and 6 (CDK4/6) are serine–threonine kinases with a key role cell cycle progression from the G1 to the S phase. Once interaction with D-type cyclins is activated, CDK4/6 phosphorylates retinoblastoma protein 1 (RB1), an oncosuppressor, the primary function of which is to inhibit the transcriptional factors of the E2F family, preventing unchecked cell cycle progression and proliferation. The functional inactivation of RB1 through phosphorylation allows for the release of E2Fs and the transcription of genes required for S-phase entry, thus triggering the cell cycle cascade [1,2].

The discovery that dysregulation of the CDK4/6 pathway is involved in breast cancer (BC) cell proliferation [3] has led to the development of selective CDK4/6 inhibitors, which have dramatically changed the management of breast cancer (BC) patients in advanced settings [4]. Three CDK4/6 inhibitors, namely palbociclib, abemaciclib, and ribociclib, are currently approved as treatment of hormone-receptor-positive (HR+), human epidermal growth factor receptor 2-negative (HER2-) advanced BC in combination with endocrine therapy (ET) [5]. The approval of these new agents was based on the results of several phase III clinical trials, which were consistent in reporting an overall survival (OS) improvement with ribociclib and abemaciclib and an approximate doubling of progression-free survival (PFS) with each of these three agents in BC patients treated with CDK4/6 inhibitors plus ET compared with ET plus placebo. Favorable results were observed both in the first- and second-line settings (PALOMA-2 for palbociclib plus letrozole; MONALEESA-2 for ribociclib plus letrozole; MONARCH-3 for abemaciclib plus letrozole or anastrozole; PALOMA-3 for palbociclib plus fulvestrant [6]; MONARCH-2 for abemaciclib and fulvestrant; and MONALEESA-3 for ribociclib plus fulvestrant) [6,7,8,9,10,11]. The revolutionary improvement in clinical outcomes conferred by CDK4/6 inhibitors in HR+/HER2- BC has fueled the design of preclinical [12,13] and early-phase clinical [14] studies investigating their activity in the triple-negative BC (TNBC) subtype, with early results suggesting their potential use in some patient subgroups.

Despite the remarkable results obtained by CDK4/6 inhibitors, approximately 10% of patients exhibit primary resistance, whereas acquired resistance inevitably occurs, leading to treatment failure [15]. Resistance mechanisms may differ depending on the type of inhibitor due to the variable target sensitivity and the distinct transcriptional, proteomic, and phenotypic effects observed following treatment with palbociclib, abemaciclib, and ribociclib [15]. Moreover, differences in terms of metastatic site/s, disease-free survival (DFS, in patients from the adjuvant setting), and therapy/ies administered prior to CDK4/6 inhibitors may affect the response to treatment and the onset of resistance. Resistance against CDK4/6 inhibitors is not exclusively driven by genomic aberrations exemplified by point mutations, insertions/deletions, or amplifications but also by changes in gene expression. The identification of differentially expressed genes or gene signatures able to predict efficacy/effectiveness or resistance to CDK4/6 inhibitors is critical in medical decision making. A key aspect also relates to the predictive role of the biomarkers of interest in terms of treatment toxicity, which may severely affect patient compliance and significantly impact treatment outcomes. Research on biomarkers holds the potentials to unveil targets for molecularly driven treatments to avoid or counteract primary or acquired resistance against CDK4/6 inhibitors [16]. To date, several biomarkers involved in CDK4/6 resistance have been identified, including cell cycle mediators, genes participating in oncogenic kinase pathways [16,17], and genes regulating the tumor microenvironment, which have been shown to clearly influence the antitumor response elicited by CDK4/6 inhibitors [18].

In this review, we summarize the most representative and updated evidence on biomarkers predictive of response/resistance to CDK4/6 inhibitors. To this end, we first address the main mechanisms of resistance against CDK4/6 inhibitors and highlight recent advances in biomarker identification. Special focus is placed on the potential use of liquid biopsy in the optimization of decision making in BC patient management and on the role of multiple microRNAs (miRNAs) as molecular predictors of treatment response/resistance to CDK4/6 inhibitors.

## 2. Mechanisms of Resistance to CDK4/6 Inhibitors

**Figure 1:**
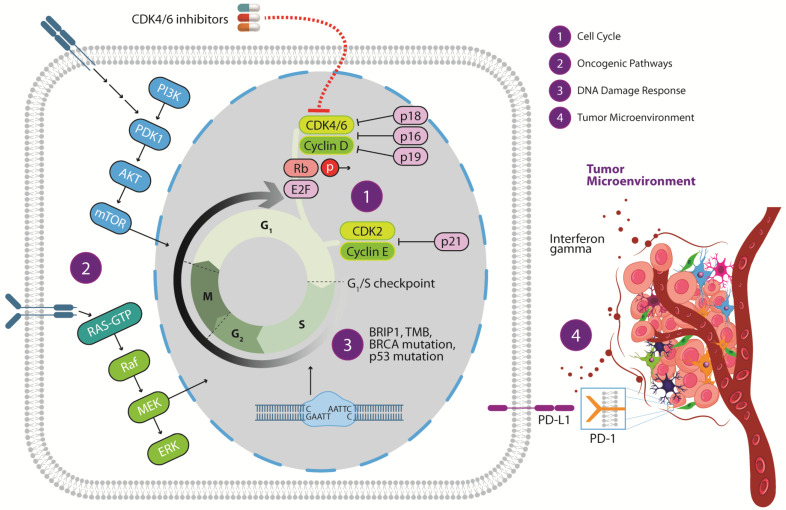
Mechanisms of resistance to CDK4/6 inhibitors. (1) Mechanisms related to the cell cycle; (2) oncogenic signaling pathways; (3) DNA damage response and repair deficiency; (4) mechanisms related to the microenvironment. PI3K: phosphoinositide 3-kinase; PDK1: 3-phosphoinositide-dependent protein kinase 1; AKT: protein kinase B; mTOR: mammalian target of rapamycin; RAS-GTP: RAS proto-oncogene GTPase; RAF: RAF proto-oncogene serine/threonine protein kinase; MEK: Mitogen-activated protein kinase; ERK: extracellular signal-regulated kinases; Rb: RB transcriptional corepressor; p16: cyclin-dependent kinase inhibitor 2A; p18: cyclin-dependent kinase inhibitor 2C; p19: cyclin-dependent kinase inhibitor 2; E2F: E2F transcription factor; CDK: cyclin-dependent kinase; p21: cyclin-dependent kinase inhibitor 1A; BRIP1: BRCA1 interacting helicase 1; *BRCA*: breast cancer gene; HRR: homologous related repair; PD-1: programmed cell death protein 1; PD-L1: programmed cell death protein 1 ligand; TMB: tumor mutational burden.

The main resistance mechanisms to CDK4/6 are discussed below and graphically displayed in Figure 1.

### 2.1. Cell Cycle Mediators as Resistance Biomarkers

As mentioned above, inhibition of CDK4 and CDK6 leads to hypophosphorylation of RB1 and its family members. p130 and p107, resulting in binding and repression of transcription factor E2F, which is required for cell cycle progression [1,2]. Cyclin E1 is the most prominent factor identified as upregulated in resistant BC [19,20,21]. Overexpression of cyclin E1 leads to the activation and rewiring of CDK2, which enables the cell to bypass the cyclin D1-CDK4/6 blockade of RB1 and to enter a non-canonical S phase [22]. High expression of CDK6 has also been reported to play an important role in the resistance mechanism [23]. Increased expression of the cyclin-dependent kinase inhibitors 2D (CDKN2D, p19) and 2C (CDKN2C, p18), which belong to the INK4 family, has also been associated with reduced efficacy of CDK4/6 inhibitors plus ET [19,20], suggesting that these tumors may have already lost their dependency on the CDK4/6 restriction point. In addition, upregulation of p16 (CDKN2A) at the protein level [24] and an increase in the expression of E2F targets and other cell-cycle-related pathways, including Myc regulation [19], has been reported in patients with resistant BC, highlighting the critical role of these genes in the clinical efficacy of CDK4/6 [19,20].

### 2.2. Oncogenic Signaling Pathways Promoting CDK4/6 Inhibitor Resistance

Further upregulated gene signatures, such as the mammalian target of rapamycin complex 1 (mTORC1), have been identified as being related to reduced CDK4/6 inhibitor response [19]. Recognition of the importance of the deregulated PI3K/AKT/mTOR pathway has given rise to ongoing clinical trials of combination therapies targeting mTOR or upstream kinases of mTOR, such as AKT1 or PI3K [16].

Activation of fibroblast growth factor receptors (FGFRs) throughout gene amplification and/or increased mRNA expression are associated with shorter PFS following treatment with CDK4/6 inhibitors and ET [25]. Amplifications and overexpression of the AURKA kinase have also been reported in patients resistant to CDK4/6 inhibitors [18,26], similarly to amplifications or mutations leading to the activation of the RAS pathway, which drives CDK4/6 inhibitor resistance [27]. Clinical trials targeting these kinase pathways in combination with CDK4/6 inhibitors are currently ongoing to assess the efficacy of a combined approach in delaying or counteracting the insurgence of resistance [16].

### 2.3. The Role of DNA Damage Response and Repair Deficiency in the Promotion of CDK4/6 Inhibitor Resistance

Breast cancer is closely related to DNA damage response and repair (DDR) defects, which allow cells carrying altered genomic information to undergo cell division and transmit mutations to daughter cells [28].

An extensive gene expression profiling of baseline tumor tissue from patients enrolled in the PALOMA-3 trial, which randomized MBC HR+/HER2- patients with endocrine resistant disease to receive palbociclib + fulvestrant versus fulvestrant alone, showed that among the 50 hallmark gene sets from the Molecular Signatures Database, DDR was among the most significantly involved pathways in resistance to palbociclib [19]. These generic clues were subsequently supported in other investigations. Lee et al. conducted an exploratory analysis of Young Pearl phase II trial patients allocated in the palbociclib + exemestane treatment arm by performing targeted sequencing on baseline tumor samples. The results of this investigation showed that mutational alterations of DDR components had a significant impact on PFS under palbociclib + exemestane treatment. In particular, a shorter PFS was associated with mutations/copy number alterations in the following genes: *TP53*, *BRCA2*, *ATM*, *CHEK1*, *BRIP1*, and *RAD51C*. Besides the single-gene mutations, shorter PFS was also recorded in the case of homologous related repair (HRR), as well as loss of function and increased tumor mutational burden (TMB), which are both strictly related to DDR deficiency [18]. Moreover, in a retrospective study, Lee et al. performed tumor tissue genomic and transcriptomic profiling in a cohort of patients with HR+/HER2- MBC treated with palbociclib, ribociclib, or abemaciclib and hormone therapy in different lines. Mutations in key genes involved in DDR, such as *PALB2, BRCA1*, and *ATRX*, were associated with worse PFS [29].

### 2.4. The Role of the Microenvironment in the CDK4/6 Inhibitor Response

The tumor microenvironment plays an important role in the efficacy of CDK4/6 inhibitors, which have been proven to promote changes in the tumor status through the activation of antitumor immunity. In preclinical studies, abemaciclib suppressed the proliferation of regulatory T cells (T regs) and induced the expression of antigen processing and presentation genes [30,31]. Moreover, small-molecule screening identified CDK4/6 inhibitors as compounds capable of enhancing T-cell activity and tumor infiltration [32]. Preclinical studies in mice also demonstrated that CDK4/6 inhibitors augmented the recruitment of exogenous T cells into the tumor tissue, suggesting that CDK4/6 inhibitors may sensitize patients to T-cell-delivering therapies, such as adoptive cell transfer of T cells and chimeric antigen receptors (CAR) T cells [33].

Furthermore, CDK4/6 inhibitors have been shown to decrease the stability and thus the protein level of the programmed cell death ligand 1 (PD-L1), an immune inhibitory receptor ligand that acts, together with its receptor PD1, to block T-cell activation [34]. Gene expression data from the NeoPalAna and neoMONARCH trials revealed an upregulation of gene signatures related to “allograft rejection”, “inflammatory response”, and “interferon-gamma response” in BC tissue after palbociclib and abemaciclib treatment [21,30]. On the other hand, in the PALOMA-2 study, high expression of the immunosuppressive factor PD-1 was associated with shorter PFS [7]. In the Young Pearl study, low interferon and high T-reg expression also showed a trend toward a worse prognosis after palbociclib and ET. However, the number of patients was too low for statistical significance [18]. Schaer et al. conducted a preclinical in vivo study with a phased treatment schedule of abemaciclib and anti-PDL-1 therapy, which exerted a synergistic antitumor effect by enhancing the expression of genes indicative of T-cell-mediated immune activation and inflammation, increasing the downregulation of cell cycle genes and fostering the development of immunological memory against tumor cells [35].

Based on these findings, current clinical trials are investigating the combination of CDK4/6 inhibitors and immune checkpoint inhibitors [16]; this strategy may increase the response to immunotherapy in estrogen-receptor-positive (ER+) BC, which showed only minimal response to the use of checkpoint inhibitors as single agents [36].

## 3. The Use of Liquid Biopsy for Biomarker Identification

Biomarker identification and molecular characterization of tumors play an essential role in the management of invasive BC. According to previous studies, to optimize the predictive value of molecular profiling, the latter should be ideally performed on cancer tissue from a metastatic site amenable to biopsy rather than from the primary cancer site, owing to the genomic evolution and acquisition of driver mutations over the course of cancer progression and metastatic spread [19]. However, for a considerable percentage of patients with advanced BC, only small specimens are available from metastases. This imposes a significant barrier to biomarker testing, and given the limited size of the sampled tissue, inference ability is limited concerning the representation of intratumor heterogeneity [37]. In recent years, the use of liquid biopsy in BC management has attracted increased prominence as a valid alternative to the gold standard of tissue analysis. In addition, liquid biopsy has relevant potential for the improvement of aspects of early diagnosis and screening, prediction of prognosis, monitoring of disease progression, and response to treatment [38].

Liquid biopsies allow for isolation of components of tumor cells released into blood plasma or cerebrospinal fluid, some of which include circulating tumor cells (CTCs); circulating tumor DNA (ctDNA); cell-free RNA (mRNA and miRNA); and microvesicles, including exosomes and tumor “educated platelets” [39,40,41].

### 3.1. Circulating Tumor DNA

Circulating tumor DNA (ct-DNA) is single- or double-stranded DNA released from tumor cells into the bloodstream, therefore harboring tumor-derived genetic and epigenetic features [42]. ct-DNA has been assessed as a novel biomarker for liquid biopsy in cancer diagnosis and prognosis, offering an alternative to invasive tissue biopsies [43]. Next-generation sequencing (NGS) can detect low levels of ct-DNA in the blood plasma and target a single gene or a subset of genes or identify genome-wide tumor-derived alterations in ct-DNA. Recent studies carried out through the analysis of ct-DNA released in the plasma of patients with ER+HER2- advanced BC identified putative predictive biomarkers of resistance to CDK4/6 inhibitors in ct-DNA, showing that ct-DNA analysis can be used for biomarker detection to inform genotyping-guided precision medicine [44]. The retinoblastoma gene 1 (*RB1*) is a putative biomarker for BC treatment, as its genomic alteration appears to be associated with CDK4/6 inhibitor resistance [45,46]; in the PALOMA-3 trial, loss of RB1 detected by basally derived ct-DNA from 195 patients was associated with worse PFS in patients treated with palbociclib and fulvestrant [41]. Moreover, Condorelli and colleagues showed, through a ct-DNA analysis, that loss of function of *RB1* after CDK4/6 inhibitor treatment (palbociclib/ribociclib) was associated with acquired CDK4/6 inhibitor resistance in patients with metastatic BC [46].

The estrogen receptor alpha gene (*ESR1*) could also represent a relevant biomarker of CDK4/6 therapy resistance. However, its role remains poorly clarified, and further studies are needed to investigate its status in BC treatment [45,47]. Driver mutations in *ESR1* were reported in 25% of patients in the PALOMA-3 trial who underwent ct-DNA analysis (N = 331; 63.5%). Patients with tumors harboring *ESR1* mutations had a worse median PFS only in the placebo arm, whereas palbociclib was found to provide similar effects, regardless of *ESR1* mutations [45]. Different results were observed in MONARCH-2, in which patients with *ERS1* mutations showed an overall survival benefit [47].

The potential predictive role of *KRAS*, a gene coding for an oncoprotein that acts upstream of CDK4/6 by modulating cyclin D1 overexpression [3], has also been analyzed for CDK4/6 inhibitor resistance in patients with metastatic BC. The authors investigated *KRAS* status in the ctDNA of 106 patients with HR+HER2- advanced BC treated with palbociclib in combination with fulvestrant. The study showed that *KRAS* mutations were significantly associated with resistance to palbociclib, worse PFS, and tumor relapses within approximately 6 months [48].

Other genomic mutations have been investigated in plasma ct-DNA of patients with BC treated with CDK4/6 inhibitors, such as *PIK3CA*, *TP53*, *MYC*, *CCND1*, *CDK4*, *CDKN1*, *CDKN2*, *NF1,* and *ERBB2* [45,49]. In particular, a recent study showed that patients with *CCND1* alterations in ct-DNA at baseline had a worse median PFS after ribociclib treatment, suggesting a role of *CCND1* as a prognostic biomarker. The authors also reported that gene alterations in *MYC* and *TP53* correlated with worse PFS, although ribociclib benefit was independent of mutations in *MYC* and *TP53* [49].

### 3.2. Circulating Free RNA

A further important component of tumor cells detectable through liquid biopsy is cell-free RNA (cf-RNA), which is produced in large amounts by tumor cells, owing to their high turnover rate, and is released into the bloodstream in the form of ribonucleoprotein complexes, platelets, or CTCs and extracellular vesicles, such as exosomes [40]. Del Re and coauthors reported that high baseline CDK4 mRNA quantities in plasma-derived exosomes were correlated with response to palbociclib plus ET. Conversely, increased thymidine kinase 1 (TK1) and CDK9 mRNA were associated with clinical resistance [50]. Similar results were reported by Bagegni and colleagues, who showed that increased serum TK1 activity was significantly associated with tumor cell proliferation response in patients with early-stage HR+ HER2- BC. These results were observed as early as two weeks after the start of CDK4/6 therapy, showing that TK1 is a promising pharmacodynamic marker of palbociclib and that liquid biopsy may be an optimal tool for non-invasive early detection and longitudinal monitoring of patient response [40,51].

## 4. MiRNAs as a Tool for Response Prediction to CDK4/6 Inhibitors

MiRNAs are small non-coding RNAs consisting of 20–22 nucleotides, which play a fundamental role in development and tumorigenesis. These molecules act at the post-transcriptional level by negatively regulating genes involved in several processes related to proliferation, development, differentiation, apoptosis, and cancer. Notably, they bind by base pairing the 3′ untranslated region (3′UTR) of the mRNA targets, either inhibiting their translation or causing their destabilization and thus degradation [52].

In recent decades, multiple research projects have focused on the role of miRNAs as predictive biomarkers, as their deregulation is often associated with tumor progression [53]. miRNAs can be divided into oncogenic miRNAs (oncomirs) and oncosuppressor miRNAs; the former support cancer progression by indirectly or directly controlling the expression of genes related to increased cell proliferation, tumor development, and metastases, whereas the latter are involved in the negative regulation of oncosuppressor genes that are crucial to prevent a cancer-prone landscape [54]. Considering the peculiar ability of a single miRNA to bind and control hundreds of mRNA targets and that different miRNAs can regulate each target, miRNA deregulation can be monitored and targeted according to a precise personalized medicine approach. Interestingly, owing to their resistance and presence in body fluids, miRNAs could play a role as pivotal circulating biomarkers detectable in liquid biopsy to help monitor patient response to therapy and as targets to prevent cancer development [40].

The discovery of miRNAs is relatively more recent than other biomarkers. However, multiple preclinical studies have already investigated the role of miRNAs in modulating response or resistance to CDK inhibitors, providing evidence that miRNAs could potentially serve as predictive biomarkers for treatment with CDK4/6 inhibitors and that target therapies using specific miRNAs could potentially maximize sensitivity to these agents [55]. Overall, literature findings suggest that some miRNAs (such as miR-326, miR-29b-3p, miR-126, and miR3613-3p) are associated with sensitivity to CDK4/6 inhibitors, whereas others (such as miR-432-5p, miR-223, and miR-106b) appear to confer treatment resistance. The main results of studies on miRNAs with respect to CDK4/6 inhibitors are briefly discussed below and summarized in Table 1.

### 4.1. miRNAs Promoting CDK 4/6 Inhibitors Sensitivity

miR-326: Very low levels of miR-326 were detected in more aggressive BC cells compared with less aggressive types [62]. Shang A. and colleagues reported that ectopic expression of miR-326 had a moderate antiproliferative effect on BC cells; the same finding was reported by Baldisseri’s group (*p* = 0.058) and was associated with increased ribociclib sensitivity in HER2+ BC cell lines [56,63].

miR-29b-3p: A study by Ji W. and colleagues unveiled a novel c-myc/miR-29b-3p/CDK6 axis in luminal and HER2+ BC cell lines [57]. miR-29b-3p exerts an antiproliferative effect by inducing cell cycle arrest and reducing epithelial–mesenchymal transition (EMT) through the downregulation of CDK6 expression in BC cells. Accordingly, the downregulation of miR-29b-3p expression was associated with resistance to the CDK 4/6 inhibitor palbociclib. Conversely, its overexpression led to the sensitization of palbociclib-resistant BC, suggesting a promising therapeutic utilization of this miRNA to sensitize cancer cells that have developed palbociclib resistance.

miR-126: The CDK 4/6 inhibitor ribociclib has shown an enhanced anti-tumor effect in combination with miR-126 transfection. This miRNA sensitizes (*p* < 0.005) luminal and HER2-positive BC cells to CDK 4/6 treatment, as its targets include genes related to cell cycle progression, mitosis, and glycolysis [56].

miR-3613-3p: Yu Y. and colleagues reported that miR-3613-3p acts as a tumor suppressor miRNA in triple-negative BC cells (TNBC), where its expression appears to be downregulated [58]. This miRNA exerts its function by directly regulating *SMAD2* and *EZH2* expression, two important genes involved in cell proliferation and cancer growth. The combination of miR-3613-3p overexpression and palbociclib enhanced the sensitivity of TNBC cells to the CDK4/6 inhibitor, inducing cell cycle arrest and triggering senescence [58].

### 4.2. miRNAs Promoting CDK 4/6 Inhibitor Resistance

miR-432-5p: Cornell L. and colleagues reported that miR-432-5p expression is increased in palbociclib-resistant ER+ BC cell lines [55]. These cells exhibit increased expression of CDK 6 and CCND1; moreover, through extracellular signaling, parental cells become resistant to palbociclib when cocultured with resistant cells. miR-432-5p suppresses SMAD4 and the TGF-β pathway by triggering CDK6 overexpression and supporting CDK 4/6 inhibitor resistance [59].

miR-223: Citron F. and colleagues reported that miR-223 is downregulated in luminal and HER2+ BC cells, where it is associated with resistance to CDK 4/6 inhibitor palbociclib [60]. During cancer onset, activation of the E2F1 transcriptional factor leads to miR-223 repression and consequently induces a reduction in overall survival rates. Citron’s group described the significance of their work, proposing miR-223 as a novel biomarker of response to CDK 4/6 inhibitor treatment [60].

miR-106b: Palbociclib treatment downregulates miR-106b expression in ER+ BC cells via the RB pathway through modulation of MCM7 RB/E2F target gene expression [51]. Interestingly, as reported by Chen X. E. and colleagues, one of the miR-106b targets is the oncosuppressor *PTEN*, which is upregulated following palbociclib treatment [64].

Based on the aforementioned evidence, identifying specific expression patterns of miRNAs could be a promising approach to study tumor response to CDK 4/6 inhibitors and exploit them as novel biomarkers. Owing to their resistance to degradation and their presence in body fluids as circulating miRNAs, they represent an innovative and powerful non-invasive tool that can be easily used and implemented, particularly in light of the recent advances in liquid biopsy [40]. In addition, unlike protein-coding genes, non-coding RNAs have been demonstrated to be strictly lineage-specific; their expression may therefore determine cell phenotype, allowing for the identification of specific tumor sub-populations resistant to CDK inhibitors [65].

## 5. CDK4/6 Inhibitors in TNBC and Additional Hints on Resistance Mechanisms

TNBC is a heterogeneous disease [66] that accounts for approximately 15% of BC cases and is characterized by increased incidence of distant metastases and poorer prognosis compared to the other BC subtypes. These features, coupled with the lack of targetable receptors (such as HR and HER2), make the treatment of TNBC very challenging in most of cases [67], denoting an urgent need for new therapeutic options. The impressive clinical benefit obtained by use of CDK4/6 inhibitors in HR+/HER2- BC, together with their mechanism of action, has made them an attractive approach to be tested for the treatment of TNBC. A proficient RB1 is mandatory for the action of CDK4/6 inhibitors, as the later exert their effect of blocking G1/S cell cycle transition by favoring an unphosphorylated state of the former. In a well-known study by Finn et al., it was demonstrated that TNBC cell lines were less sensitive to palbociclib compared to their HR+ counterpart [12]. Subsequently, a more granular investigation included single cell analysis to test the activity of palbociclib and ribociclib on 12 TNBC cell lines, including all the subgroups identified in early gene profiling studies, namely basal-like, luminal androgen receptor (AR)-like (LAR), mesenchymal (MES), and mesenchymal-stem-like (MSL) cells. Results showed that LAR cell lines were more sensitive to the agents than basal-like and MES cell lines, as also confirmed in xenograft models [13]. The best explanation for these findings is that RB1 loss, which permanently cancels E2F inhibition, is a frequent event in basal-like TNBC but not in the LAR subgroup [68]. Moreover, basal-like, MES, and MSL subgroups exhibit the typical genomic alterations associated with TNBC more frequently, which could explain CDK4/6 resistance, such as Cyclin E1 amplification, *TP53* mutation, and increased CDKN2A expression [69,70]. Sequential or combination therapies of CDK4/6 inhibitors and other agents have been investigated as possible strategies to overcome resistance or increase activity in TNBC. In particular, palbociclib pretreatment increased the sensitivity of TNBC cell lines to paclitaxel [71], whereas the treatment of AR+ TNBC cell lines concomitantly with palbociclib and enzalutamide resulted in a synergistic increase in the cytostatic effect produced by the two agents administered separately [72].

The evidence regarding the efficacy of CDK4/6 inhibitors in clinical trials is scarce. In 2015, a phase 2 clinical trial assessing the safety of palbociclib in 37 BC patients showed a shorter PFS in the 4 TNBC patients relative to the 33 patients with HR+ disease (1.5 months vs. 4.5 months). Since then, several trials have started to investigate the efficacy of CDK4/6 inhibitors in TNBC, mostly in combination with other agents. These trials are ongoing; some examples include the assessment, in early-phase trials, of combinations such as palbociclib and bicalutamide (NCT02605486), palbociclib and avelumab, (NCT04360941), and ribociclib and bicalutamide (NCT03090165). Another interesting ongoing phase 2 trial is assessing the safety of single-agent abemaciclib in RB1-positive TNBC patients (NCT03090165).

## 6. Additional Cancer Genomic Alterations Involved in Resistance to CDK4/6 Inhibitors

The spectrum of genomic alterations that impact the activity of CDK4/6 inhibitors goes beyond those mentioned in the context of liquid biopsy and those modulated by microRNAs. Some of the most relevant mutations these contexts are those concerning the following genes: *MLL3*, *GPR124*, *MAP2K4*, *CREBBP*, *AURKA*, and *PTEN*. Other genes confer resistance to CDK4/6 inhibitors after amplification, such as *ZNF703*, *FGFR1*, *MDM2*, *AURKA*, and *FRS2* [18,29]. This appears to be a particularly important aspect to be considered while searching for biomarkers in liquid biopsy and by microRNA profiling.

## 7. Discussion

Although CDK4/6 inhibitors are already a mainstay for the treatment of metastatic HR+/HER2-, there are still hurdles to overcome. The vast majority of advanced BC patients are affected by tumors that develop acquired resistance to these agents; moreover, some patients have tumors that present primary resistance, resulting in early progression. The paradigm of managing cancer patients in the context of precision medicine requires the identification and validation of biomarkers predictive of response/resistance to be implemented in clinical practice.

In this article, we report the results of a literature search designed to retrieve the most recent data on CDK4/6 inhibitor resistance mechanisms. All relevant studies that used novel methods to characterize predictive biomarkers of efficacy and resistance to these agents were included. Evidence on liquid biopsy and innovative biomarkers, such as genomic alterations, gene expression, and miRNA expression, were analyzed.

Our review of the available literature confirmed the potential of liquid biopsy in managing advanced BC treated with novel agents, similar to the results achieved by liquid biopsy when applied to other cancers and in other settings [73]. Liquid biopsy is critical for the study of cancer dynamics in a way that accounts for cancer heterogeneity, which is a key aspect in the metastatic setting that cannot be fully encompassed by the genomic or proteomic profile of a single metastatic site. On the other hand, we found that resistance to CDK4/6 inhibitors can be framed at different levels, such as the cell cycle, DNA damage, repair machinery, signaling pathways, and the tumor microenvironment. Consistent evidence has shown that ct-DNA and cf-RNA could be extracted from liquid biopsy and used to search for specific mutations and gene expression deregulation (through next-generation sequencing or classical technologies such as polymerase chain reaction) as biomarkers of resistance at all the aforementioned levels.

miRNA profiling could add another fundamental layer to the landscape of biomarker research of advanced BC treated with CDK4/6 inhibitors. Based on their mechanism of action, quantifying the relative miRNA deregulation reflects the tumor’s nature beyond its genomic and transcriptomic level, providing important insights into the altered cellular functions that are currently predominantly active. Overall, the available data confirm the multifaceted potential of these small molecules in BC, consistent with findings in other cancers [74,75].

However, concerning the specific role of miRNAs as predictors of CDK4/6 inhibitor efficacy/resistance in advanced BC, most of available evidence comes from preclinical studies conducted in BC cell lines. The deregulation of specific miRNAs seemed to affect sensitivity/resistance of BC cell lines to CDK4/6 inhibitors. Comparable results were obtained in analogous preclinical studies conducted on other tumor types. In particular, concerning miRNAs associated with increased sensitivity to CDK4/6 inhibitors, the following miRNAs and relative studies can be mentioned. The *MIR17HG* gene encodes a cluster of six miRNAs that have been shown to increase sensitivity to palbociclib in atypical teratoid rhabdoid tumors (ATRTs), a fatal pediatric malignancy of the central neural system caused by SMARCB1 deficiency [76,77]. These miRNAs negatively regulate the expression of cyclin D1, enhancing CDK4/6 inhibitor treatment efficiency. Interestingly, in proneural glioblastoma, the miRNA cluster was found to be upregulated due to E2F transcriptional factors binding to the miR-17-92 promoter. Li M. and colleagues demonstrated that glioblastoma stem-cell-like lines exhibited increased sensitivity to ribociclib and palbociclib treatment, and palbociclib treatment in combination with N, N-diethylaminobenzaldehyde (an inhibitor of the mesenchymal driver ALDH1A3) exerted a synergistic inhibitory effect on cell proliferation [78]. In another study, miR-497 was found to be downregulated in BC due to the methylation of CpG islands [79]. This miRNA acts as an oncosuppressor, as its overexpression inhibits cell proliferation and invasion. In addition, Hoareau-Aveilla C. and colleagues studied its role in NPM-Anaplastic large cell lymphoma (NPM -ALK+ ALCL) cells, where it regulates the expression of cyclin E1, CDK6, and E2F3. The authors demonstrated the sensitivity of NPM-ALK+ ALCL cells to palbociclib, suggesting that miR-497 downstream targets could be used to predict clinical outcomes [80]. Moreover, the pivotal involvement of another miRNA, miR-33a, in cell metabolism as an oncosuppressor has already been demonstrated in several types of cancer, such as lung cancer, melanoma, and BC [81,82,83,84]. Notably, Rencuzogullari O. and colleagues reported that in pancreatic cancer, palbociclib treatment also acts through the upregulation of this metabolic miRNA, regulating AMPK/mTOR signaling and enhancing pancreatic cancer cell death [85]. On the other hand, other studies have highlighted the correlation of the expression of some miRNAs with increased resistance to CDK4/6 inhibitors in different tumor types. In particular, Kauloniemi’s group demonstrated that miR-193b is hypermethylated in prostate cancer cells, leading to the upregulation of the target gene CCND1, which encodes for cyclin D1 [86]. The role of this miRNA as a tumor suppressor has also been demonstrated in other tumor types, such as non-small cell lung cancer [87] and gastric cancer [88]. Interestingly, prostate cancer cells express low levels of this miRNA and high levels of cyclin D1, supporting tumor progression. Treatment with CDK 4/6 inhibitors showed exerted an inhibiting effect on cell growth in cancer cells with low miR-193b expression. In contrast, no significant effect was observed at high miR-193b levels, high-lighting the involvement of this miRNA in CDK 4/6 inhibitor resistance [86]. Moreover, in metastatic melanoma, Bustos M. A. and colleagues reported a decrease in miR-200a expression, which was associated with resistance to palbociclib treatment [89]. As Gong’s group also reported in hepatocellular carcinoma (HCC), this miRNA directly targets CDK 6, and in melanoma cancer cells, CDK 6 downregulation induced treatment resistance [89,90]. Kim and coauthors reported that RNA-binding protein Quaking (QKI), a protein implicated in endothelial maturation and proliferation, is targeted by miR-200b in OSCC [91]. QKI plays a role in tumor angiogenesis via CCND1-mediated endothelial cell cycle progression by binding and stabilizing cyclin D1 mRNA; in turn, miR-200b influences cell cycle progression, angiogenesis, and metastasis by negatively regulating QKI expression [92]. As Azam S. H. and colleagues demonstrated, the endothelial expression of miR-200b was downregulated in lung tumor development, leading to an increase in QKI expression and the activation of the miR-200b/QKI/CCND1 pathway [92,93]. Palbociclib inhibits angiogenesis and tumor progression by disrupting the miR-200b/QKI/CCND1 axis, leading to CDK 4/6-cyclin D1 complex disruption [89]. Finally, CDK6 is a common downstream target of let-7a and miR-21. Several published articles have reported that miR-21 negatively regulates CDK6 [94,95]. Gary J.M. and colleagues observed the existence of an mTOR-let7a/miR-21/CDK 6 axis in thymic T-cell acute lymphoblastic leukemia/lymphoma (T-ALL/LBL). Following mTOR inhibition, let-7a and miR-21 expression levels were increased, leading to the downregulation of CDK6 and the triggering of palbociclib treatment resistance. Interestingly, the combination of the mTOR inhibition pathway and palbociclib exhibited an enhanced antitumor effect relative to the single treatments alone [96].

Overall, the evidence shows that investigation of miRNAs as predictive biomarkers of CDK4/6 activity in BC and other cancer types is at an embryonic stage. This highlights the urgent need to design new studies to fully exploit the potential of miRNAs in this context. Moreover, miRNA profiling can be imbricated into the already significant role of liquid-phase biopsy [62], as these small molecules can also be detected in blood and other body fluids. Several studies have shown the miRNA profiling in liquid biopsy, as single biomarkers, in the form of signatures or combinations with genomic alterations and mRNA expression levels, can undoubtedly increase the accuracy of liquid-biopsy-based techniques aimed at early diagnosis of cancer relapse, tumor burden quantification, treatment tailoring, and efficacy monitoring [97,98,99].

Altogether, this rapidly evolving evidence on the usefulness of liquid biopsy and miRNAs in cancer management stimulated our research group to design and conduct a prospective observational study, which is nearing completion, based on the idea of performing miRNA profiling in liquid biopsies and quantifying tissue-level gene expression in BC patients treated with CDK4/6 inhibitors.

Finally, an additional aspect related to miRNAs deserves mention in this literature review, especially in the milieu of methods seeking to achieve efficacy prediction during treatment. In particular, miRNAs are gaining attention for their potential use at the therapeutic level [100]. This might offer new possibilities in the future, especially in areas such as CDK4/6 treatment, where efficacy/resistance prediction could be converted into synergistic combinations of these drugs with exogenous miRNAs to increase benefits from treatment.

## 8. Conclusions

In conclusion, the outstanding efficacy of CDK4/6 inhibitors in the treatment of advanced HR+/HER2- BC highlights the need to develop molecular biomarkers that may be able to predict efficacy and/or resistance and provide a prognostic stratification of patients. We developed this review with these facts in mind and focused our literature search on advanced techniques, such as liquid biopsy, and novel molecular biomarkers, such as miRNAs. Data showed that the utility of liquid biopsy in the context of CDK4/6 inhibitor efficacy/resistance prediction is currently appraised at an adequate level, with several genomic and transcriptomic biomarkers already discovered. Conversely, the investigation of miRNAs in the same context appears to be in a more initial stage but with evidence confirming their potential. These data prompted our study group to develop a prospective observational study based on miRNA profiling in liquid biopsies and tumor tissue gene expression quantification in patients receiving CDK4/6 inhibitors, which is nearing completion.

Future translational studies with precise clinical and patient molecular stratifications are urgently needed and will shed light on similarities and differences in resistance mechanisms useful for defining and exploiting vulnerabilities for possible future combination therapies.

## Figures and Tables

**Table 1:** Summary of studies on miRNA predictors for CDK4/6 inhibitor (I) response or resistance.

miRNA	Tumor	CDK4/6I	Deregulation	Effect	Reference
CDK4/6I SENSITIVITY					
miR-326	HER2+ BC	Ribociclib	Upregulation	Increased ribociclib sensitivity (antiproliferation activity on BC cells)	[56]
miR-29b-3p	Luminal, HER2+ BC	Palbociclib	Upregulation (c-myc)	Sensitization to palbociclib (reduces epithelial–mesenchymal transition)	[57]
miR-126	Lumina, HER2+ BC	Ribociclib	Upregulation	Sensitization to palbociclib (targets cell cycle genes)	[56]
miR-3613-3p	TNBC	Palbociclib	Upregulation	Sensitization to palbociclib (regulates *SMAD2* and *EZH2* expression)	[58]
CDK4/6I resistance					
miR-432-5p	ER+ BC	Palbociclib Ribociclib	Upregulation	Resistance to palbociclib (suppresses SMAD4 and TGF-β pathway)	[59]
miR-223	Luminal, HER2+ BC	Palbociclib	Downregulation	Resistance to palbociclib (associated with activation of E2F1)	[60]
miR-106b	ER+ BC	Palbociclib	Downregulation	Resistance to palbociclib (modulation of MCM7 RB/E2F expression)	[61]

Abbreviations: BC: breast cancer; CDK: cyclin-dependent kinase; EGF: epidermal growth factor; EMT: epithelial–mesenchymal transition; ER+: estrogen-receptor-positive; EZH2: enhancer of zeste homolog 2; HER2+: human epidermal growth factor receptor 2 positive; MRP-1: multidrug resistance-associated protein 1; PTEN: phosphatase and tensin homolog; RB: retinoblastoma protein; SMAD: SMAD family member; TGF-β: transforming growth factor beta; TNBC: triple-negative breast cancer.

## Data Availability

Not applicable.
